# A new species of the bee genus *Ctenoplectrella* in middle Eocene Baltic amber (Hymenoptera, Megachilidae)

**DOI:** 10.3897/zookeys.111.1593

**Published:** 2011-06-22

**Authors:** Victor H. Gonzalez, Michael S. Engel

**Affiliations:** 1 Department of Ecology & Evolutionary Biology, 1200 Sunnyside Avenue, Haworth Hall, University of Kansas, Lawrence, Kansas 66045, USA; Current address: USDA-ARS Bee Biology & Systematics Laboratory, Utah State University, Logan, Utah 84322-5310, USA; 2 Division of Entomology (Paleoentomology), Natural History Museum, and Department of Ecology & Evolutionary Biology, 1501 Crestline Drive – Suite 140, University of Kansas, Lawrence, Kansas 66045, USA

**Keywords:** Megachilinae, Ctenoplectrellini, paleontology, Tertiary, Eocene, taxonomy

## Abstract

A new species of the extinct bee genus *Ctenoplectrella* Cockerell (Megachilinae: Ctenoplectrellini) is described and figured from two females preserved in middle Eocene (Lutetian) Baltic amber. *Ctenoplectrella phaeton* **sp. n.** is distinguished from its congeners on the basis of its body proportions, integumental sculpturing, wing venation, and pubescence, and is one of the more distinctive members of the genus. A revised key to the species of *Ctenoplectrella* is provided.

## Introduction

The Eocene was the last epoch harboring a truly disparate bee fauna relative to the composition of forms we are so familiar with in today’s ecosystems. Following the Eocene-Oligocene transition the bee fauna began to look relatively modern at least in terms of the general appearance of the tribes, genera, and subgenera comprising the diversity in the latest Paleogene and Neogene periods. However, from the Eocene and earlier epochs we find regularly taxa that harbor unique combinations of traits that render them challenging to fit amongst their modern counterparts even at higher taxonomic levels, alongside otherwise more modern forms (Engel 1998, 2001, 2004, 2008, 2011, unpubl. data; Wappler and Engel 2003; Engel and Perkovsky 2006; Ohl and Engel 2007; Patiny et al. 2007; Michez et al. 2007, 2009, in press; Wedmann et al. 2009). Those glimpses into the Early Paleogene highlight a diversity of genera and tribes quite distinct, exhibiting not only plesiomorphic features relative to species of today but obviously with unique adaptations and apomorphies which did not persist to the present (e.g., the bizarre facial morphologies of species of *Succinapis* Engel: *vide* Engel 2001). Our knowledge of these remarkable bees continues to grow, mostly from European deposits but now also from distant biogeographic regions such as India (Rust et al. 2010; Engel unpubl. data).

Herein we describe a recently recognized new species of the Eocene bee genus *Ctenoplectrella* (Cockerell (1909a, 1909b). The genus was described originally alongside the genus *Glyptapis* Cockerell in a distinct subfamily, Glyptapinae, which Cockerell (1909b) considered to be “near the stem-form of the Megachilidae” but simultaneously indicated that he believed their closest modern relative to be *Ctenoplectra* Smith (hence the name he chose for the group of fossil species considered herein). Cockerell’s assertion is difficult to understand given that there is actually little morphological similarity between *Ctenoplectrella* and *Ctenoplectra*, today recognized to be a distinct lineage of Apidae, and even less so between *Glyptapis* and the latter. Engel (2001) provided the first revision of the Eocene bee fauna and recognized *Ctenoplectrella* and *Glyptapis* as definitive megachilids, and at first placed them within the Osmiini, later elevating them both to tribal status alongside the other megachilines (Engel 2005). All four *Glyptapis* species, as well as the known five species of *Ctenoplectrella*, are from Baltic amber (middle Eocene) except for *Ctenoplectrella zherikhini* Engel and Perkovsky, which is known from the Late Eocene amber of the Rovno region of the Ukraine (Table 1).

**Table 1.** Currently included species in *Ctenoplectrella*.

Species	References
Baltic Amber (Lutetian)	
*Ctenoplectrella cockerelli* Engel, 2001	Engel 2001
*Ctenoplectrella gorskii* Engel, 2008	Engel 2008
*Ctenoplectrella grimaldii* Engel, 2001	Engel 2001
*Ctenoplectrella phaeton* Gonzalez & Engel, sp. n.	Present study
*Ctenoplectrella viridiceps* Cockerell, 1909a	Cockerell 1909a, 1909b; Engel 2001
Rovno Amber (Bartonian-Priabonian?)	
*Ctenoplectrella zherikhini* Engel & Perkovsky, 2006	Engel and Perkovsky 2006

While *Ctenoplectrella* and *Glyptapis* are definite oddities, we have noted a considerable similarity between *Ctenoplectrella* and the rare living genus *Aspidosmia* Brauns [presently in Anthidiini (*vide* Michener 2007), but apparently more basally related among the Megachilinae (Litman et al. in press)], with two species in southern Africa (Brauns 1926; Peters 1972). Both genera share a metatibia with relatively long setae suggesting a scopa and a forewing with a rather elongate prestigma and basal vein strongly arcuate, meeting Cu orthogonally (i.e., at a right angle), and it is likely that they are closely related, with *Aspidosmia* perhaps representing the sole survivors of the ctenoplectrelline lineage. Naturally, this conclusion requires rigorous cladistic testing but is tantalizing given the number of biogeographic connections between the sub-Saharan or East Asian fauna and extinct taxa from the European Paleogene (e.g., Ander 1942; Petrunkevitch 1958; Larsson 1978; Lourenço and Weitschat 1996; Böhme and Weitschat 1998; Engel 2001; Liu and Engel 2010; Weitschat and Wichard 2010).

## Material and methods

Morphological terminology follows that of Engel (2001) and Michener (2007), while the format for the description generally follows that used by (Engel (2001, 2008) and Engel and Perkovsky (2006). Photomicrographs were prepared using a Nikon D1x digital camera attached to an Infinity K-2 long-distance microscopic lens. Measurements were made with an ocular micrometer attached to an Olympus SZX-12 stereomicroscope and are provided for the holotype, with those of the paratype in parentheses.

## Systematic paleontology

### Tribe Ctenoplectrellini Engel, 2001 Genus Ctenoplectrella Cockerell, 1909a

#### Ctenoplectrella phaeton sp. n.

urn:lsid:zoobank.org:act:0027DEF4-DC4A-46C2-87C4-473120D5BB80

http://species-id.net/wiki/Ctenoplectrella_phaeton

Figs 1 4

##### Holotype.

 ♀, AMNH Ba-JVe-161, Baltic amber, middle Eocene (Lutetian). Deposited in the Amber Fossil Collection, Division of Invertebrate Zoology (Entomology), American Museum of Natural History, New York.

##### Paratype.

**Figure 1.**
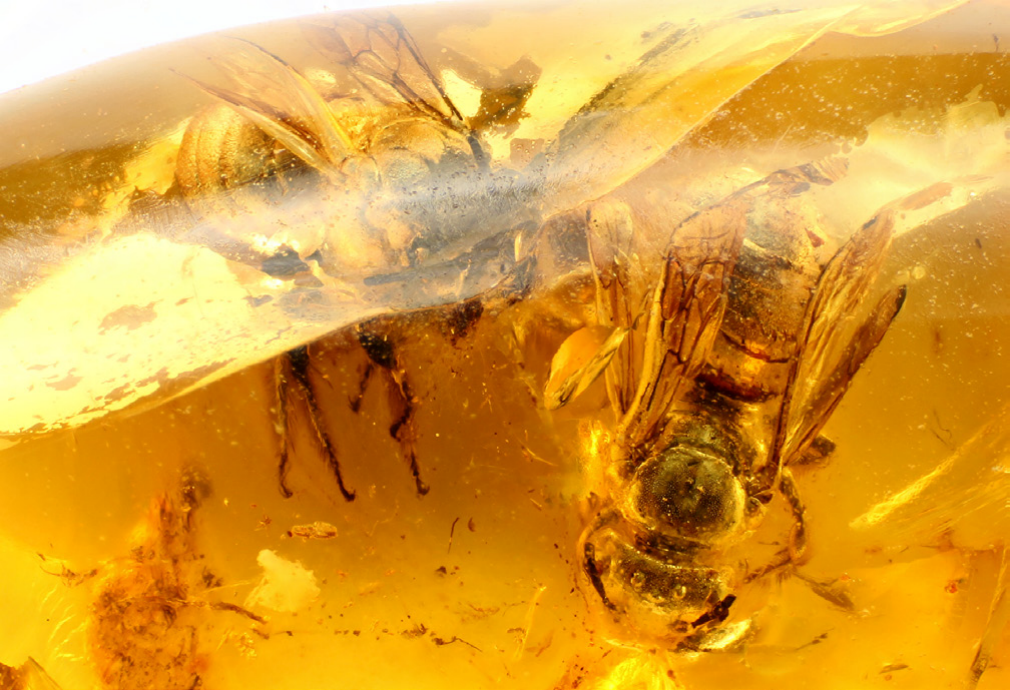
Photograph of majority of amber piece (middle Eocene, Baltic amber) indicating relative positions of two individuals of *Ctenoplectrella phaeton* Gonzalez and Engel, sp. n. (AMNH Ba-JVe-161); holotype is at right, paratype at upper left on curved edge of piece.

 ♀, on curved edge in same amber piece as holotype and with same repository (Figs 1, 4).

##### Diagnosis.

 This species resembles *Ctenoplectrella cockerelli* Engel in the forewing with vein 2rs-m strongly and doubly arcuate, the basal vein confluent with cu-a, the first submarginal cell shorter than the second submarginal cell, and the punctate mesepisternum and terga. However, *Ctenoplectrella phaeton* can be distinguished from *Ctenoplectrella cockerelli* and remaining species of the genus by its robust body, punctate metepisternum (impunctate in *Ctenoplectrella cockerelli*), and much shorter and sparser body pubescence.

##### Description.

*Female*: Total body length 5.77 mm (6.15 mm); forewing length 3.85 mm (3.92 mm). Head slightly wider than long; paraocular carina present; pedicel about as long as combined lengths of first and second flagellomeres; interocellar distance 2.5 times median ocellar diameter, 1.5 times longer than ocellocular distance; ocelloccipital distance about 1.6 times median ocellar diameter. Intertegular distance 1.46 mm. Outer surfaces of pro– and mesotibiae apically with small posterior spine. Prestigma relatively short, slightly more than two times longer than broad (prestigma width measured to its margin); basal vein strongly arcuate, confluent with cu-a; second abscissa of Rs basad 1m-cu by about six times vein width; 2rs-m distad 2m-cu by vein width, 2rs-m doubly arcuate; second submarginal cell slightly longer than first submarginal cell; seven distal hamuli, arranged in a single, evenly-spaced series. Sixth metasomal sternum with broadly rounded apical margin.

**Figures 2–4.**
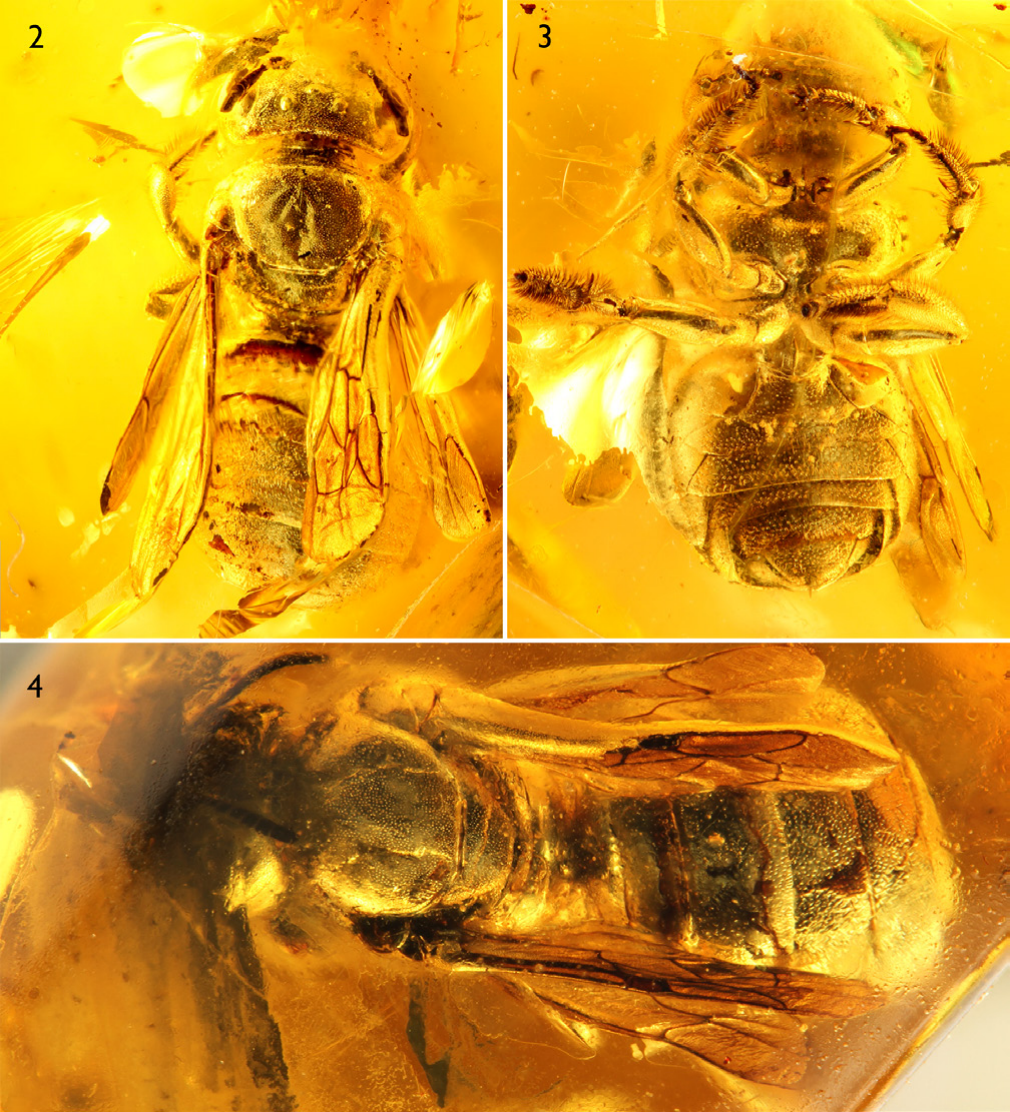
Photomicrographs of *Ctenoplectrella phaeton* Gonzalez and Engel, sp. n. (AMNH Ba-JVe-161) in middle Eocene Baltic amber. **2** Dorsal aspect of holotype female **3** Ventral aspect of holotype female **4** Dorsal aspect of paratype female.

Integument in general smooth and shiny between punctures, weakly imbricate laterally on terga. Outer surface of mandible with minute punctures separated by a puncture width or less. Frons with small punctures separated by 1–1.5 times a puncture width, punctures becoming denser towards vertex. Pronotum laterally with minute punctures separated by a puncture width or less. Mesoscutum with small punctures separated by 1–2 times a puncture width (Fig. 2); tegula with minute, scattered punctures; mesoscutellum about as punctate as on mesoscutum. Metanotum impunctate and smooth. Mesepisternum with faint, scattered, larger punctures than on mesoscutum, nearly impunctate anteriorly to omaulus, punctures denser ventrally. Metepisternum more densely punctate than on mesepisternum, punctures separated by a puncture width or less dorsally, punctures sparse ventrally. Propodeum impunctate basally, lateral and posterior surfaces with minute punctures separated by more than two times a puncture width. Metasomal terga with small punctures separated by 1–2 times a puncture width, without distinct depressed marginal zones; sterna with coarser punctures than on terga, punctures smaller and finer on first sternum.

Color apparently brown, without maculations. Wing membrane hyaline; veins strong and dark brown.

Face with minute, appressed, simple setae not obscuring integument. Mesoscutum and mesoscutellum with scattered, short, simple setae. Mesepisternum with scattered, erect, longer setae (0.5 times median ocellar diameter) than on mesoscutum. Basal area of propodeum without pubescence; lateral and posterior surfaces with minute, sparse setae (integument largely visible among setae). Legs in general with short, scattered, minutely-branched setae (Fig. 3); basitarsi with denser, slightly longer setae than on tibiae; metatibia with scattered, minutely-branched setae (setal length about 1–1.5 times median ocellar diameter). Metasoma with scattered, short (≤ 0.5 times median ocellar diameter), simple, erect to suberect setae on discs; sternal scopa composed of bands of rather sparse, long (2.5–3.0 times median ocellar diameter), erect, simple setae.

*Male*: Unknown.

##### Etymology.

 The specific epithet is taken from Phaeton and treated as a noun in apposition. In Greek mythology Phaeton died when he tried to drive the chariot of the sun across the sky. Phaeton’s sisters wept and their tears turned to amber.

##### Comments.

The supraclypeus, clypeus, and mandibles are obscured by dense *Schimmel* (whitish froth of microscopic bubbles resembling mold) in the holotype and by a fracture in the amber piece in the paratype. However, the strong apical tooth and distinct outer ridge of the mandible is barely visible in the holotype, thus suggesting a similar mandibular shape as in other species of *Ctenoplectrella*.

### Revised key to species of Ctenoplectrella

(updated from Engel and Perkovsky 2006)

**Table:** 

1	Forewing 2rs-m strongly and doubly arcuate, thus second submarginal cell more strongly produced toward wing apex along posterior margin; medioapical margin of clypeus straight (shape of clypeus unknown in *Ctenoplectrella phaeton*)	2
–	Forewing 2rs-m relatively straight and therefore second submarginal cell not more strongly produced toward wing apex along posterior margin; medioapical margin of clypeus gently convex	*Ctenoplectrella viridiceps* Cockerell
2	Forewing basal vein confluent with cu-a; first submarginal cell shorter than second submarginal cell	3
–	Forewing basal vein distad cu-a; first submarginal cell longer than second submarginal cell	*Ctenoplectrella gorskii* Engel
3	Mesepisternum impunctate laterally; metasomal terga faintly imbricate	4
–	Mesepisternum with coarse, faint punctures laterally; metasomal terga with small, scattered punctures	5
4	Propodeal setae long, erect, and branched; tarsal setae fuscous; gena tapering in width from widest above to narrower below	*Ctenoplectrella zherikhini* Engel & Perkovsky
–	Propodeal setae scattered, short, and simple; tarsal setae white or off-white; gena of relatively equal width along its length	*Ctenoplectrella grimaldii* Engel
5	Metepisternum punctate; body pubescence distinctly short and sparse	*Ctenoplectrella phaeton* sp. n.
–	Metepisternum impunctate; body pubescence of moderate length, not distinctly short and sparse	*Ctenoplectrella cockerelli* Engel

## Supplementary Material

XML Treatment for Ctenoplectrella phaeton

## References

[B1] Ander K (1942) Die Insektenfauna des balitschen Bernsteins nebst damit verknüpften zoogeographischen Problemen. Lunds Universitets Årsskrift, 2 Afdelning, Medicin samt Matematiska och Naturvetenskapliga Ämnen [Acta Universitatis Lundensis] 38 (4):1-83.

[B2] Böhme W Weitschat W (1998) Redescription of the Eocene lacertid lizard *Nucras succinea* Boulenger, 1917 from Baltic amber and its allocation to *Succinilacerta* n. gen. Mitteilungen aus dem Geologisch-Paläontologischen Institut der Universität Hamburg 81:203-222.

[B3] Brauns H (1926) V. Nachtrag zu ‘Friese, Bienen Afrikas’. Zoologische Jahrbücher, Abteilung für Systematik, Geographie und Biologie der Tiere 52: 187–230, +1 pl.

[B4] Cockerell TDA (1909a) Some European fossil bees. Entomologist 42:313-317.

[B5] Cockerell TDA (1909b) Descriptions of Hymenoptera from Baltic amber. Schriften der Physikalisch-ökonomischen Gesellschaft, Königsberg 50 (1):1-20.

[B6] Engel MS (1998) A new species of the Baltic amber bee genus *Electrapis* (Hymenoptera: Apidae). Journal of Hymenoptera Research 7 (1):94-101.

[B7] Engel MS (2001) A monograph of the Baltic amber bees and evolution of the Apoidea (Hymenoptera). Bulletin of the American Museum of Natural History 259: 1–192.

[B8] Engel MS (2004) Geological history of the bees (Hymenoptera: Apoidea). Revista Tecnologia e Ambiente 10 (2):9-33.

[B9] Engel MS (2005) Family-group names for bees (Hymenoptera: Apoidea). American Museum Novitates 3476: 1–33. 10.1206/0003-0082(2005)476[0001:FNFBHA]2.0.CO;2

[B10] Engel MS (2008) A new species of *Ctenoplectrella* in Baltic amber (Hymenoptera: Megachilidae). Acta Zoologica Academiae Scientiarum Hungaricae 54 (4):319-324.

[B11] Engel MS (2011) Systematic melittology: Where to from here? Systematic Entomology 36(1): 2–15. 10.1111/j.1365-3113.2010.00544.x

[B12] Engel MS Perkovsky EE (2006) An Eocene bee in Rovno amber, Ukraine (Hymenoptera: Megachilidae). American Museum Novitates 3506: 1–12. 10.1206/0003-0082(2006)506[0001:AEBIRA]2.0.CO;2

[B13] Larsson SG (1978) Baltic amber – a palaeobiological study. Entomonograph 1:1-192.

[B14] Litman JR Danforth BN Eardley CD Praz CJ (in press) Why do leafcutter bees cut leaves? New insights into the early evolution of bees. Proceedings of the Royal Society, Series B, Biological Sciences 10.1098/rspb.2011.0365 PMC3189370 21490010

[B15] Liu Z Engel MS (2010) Baltic amber Ibaliidae (Hymenoptera: Cynipidae): A new genus with implications for the phylogeny and historical biogeography of the family. Systematic Entomology 35(1): 164–171. 10.1111/j.1365-3113.2009.00494.x

[B16] Lourenço WR Weitschat W (1996) More than 120 years after its description, the enigmatic status of the genus of the Baltic amber scorpion *Tityus eogenus* Menge, 1869 can finally be clarified. Mitteilungen aus dem Geologisch-Paläontologischen Institut der Universität Hamburg 79:183-193.

[B17] Michener CD (2007) The Bees of the World [2nd Edition]. Johns Hopkins University Press, Baltimore, xvi+[i]+953 pp., +20 pls.

[B18] Michez D Nel A Menier J-J Rasmont P (2007) The oldest fossil of a melittid bee (Hymenoptera: Apiformes) from the early Eocene of Oise (France). Zoological Journal of the Linnean Society 150(4): 701–709. 10.1111/j.1096-3642.2007.00307.x

[B19] Michez D De Meulemeester T Rasmont P Nel A Patiny S (2009) New fossil evidence of the early diversification of bees: *Paleohabropoda oudardi* from the French Paleocene (Hymenoptera, Apidae, Anthophorini). Zoologica Scripta 38(2): 171–181. 10.1111/j.1463-6409.2008.00362.x

[B20] Michez D Vanderplanck M Engel MS (in press) Fossil bees and their plant associates. In: Patiny S (Ed) Evolution of Plant-Pollinator Relationships. Cambridge University Press, Cambridge.

[B21] Ohl M Engel MS (2007) Die Fossilgeschichte der Bienen und ihrer nächsten Verwandten (Hymenoptera: Apoidea). Denisia 20:687-700.

[B22] Patiny S Engel MS Vanmarsenille P Michez D (2007) A new record of *Thaumastobombus andreniformis* Engel 2001 in Eocene amber (Hymenoptera: Apidae). Annales de la Société Entomologique de France 43 (4):505-508.

[B23] Peters DS (1972) Über die Stellung von *Aspidosmia* Brauns 1926 nebst allgemeinen Erörterungen der phylogenetischen Systematik der Megachilidae (Insecta, Hymenoptera, Apoidea). Apidologie 3 (2):167-186. 10.1051/apido:19720204

[B24] Petrunkevitch A (1958) Amber spiders in European collections. Transactions of the Connecticut Academy of Arts and Sciences 41:97-400.

[B25] Rust J Singh H Rana RS McCann T Singh L Anderson K Sarkar N Nascimbene PC Stebner F Thomas JC Solórzano-Kraemer M Williams CJ Engel MS Sahni A Grimaldi D (2010) Biogeographic and evolutionary implications of a diverse paleobiota in amber from the early Eocene of India. Proceedings of the National Academy of Sciences, U.S.A. 107(43): 18360–18365. 10.1073/pnas.1007407107 PMC2972964 20974929

[B26] Wappler T Engel MS (2003) The middle Eocene bee faunas of Eckfeld and Messel, Germany (Hymenoptera: Apoidea). Journal of Paleontology 77 (5):908-921.

[B27] Wedmann S Wappler T Engel MS (2009) Direct and indirect fossil records of megachilid bees from the Paleogene of central Europe (Hymenoptera: Megachilidae). Naturwissenschaften 96(6): 703–712. 10.1007/s00114-009-0525-x 19296064

[B28] Weitschat W Wichard W (2010) Baltic amber. In: Penney D (Ed) Biodiversity of Fossils in Amber from the Major World Deposits. Siri Scientific Press, Manchester, 80–115 [total volume 304 pp.]

